# Leveraging plant physiological dynamics using physical reservoir computing

**DOI:** 10.1038/s41598-022-16874-0

**Published:** 2022-07-22

**Authors:** Olivier Pieters, Tom De Swaef, Michiel Stock, Francis wyffels

**Affiliations:** 1 IDLAB-AIRO—Ghent University—imec, Technologiepark-Zwijnaarde 126, 9052 Zwijnaarde, Belgium; 2 Plant Sciences Unit, Flanders Research Institute for Agriculture, Fisheries and Food, Caritasstraat 39, 9090 Melle, Belgium; 3 KERMIT and Biobix, Department of Data Analysis and Mathematical Modelling, Ghent University, Coupure Links 653, 9000 Ghent, Belgium

**Keywords:** Computer science, Plant sciences

## Abstract

Plants are complex organisms subject to variable environmental conditions, which influence their physiology and phenotype dynamically. We propose to interpret plants as reservoirs in physical reservoir computing. The physical reservoir computing paradigm originates from computer science; instead of relying on Boolean circuits to perform computations, any substrate that exhibits complex non-linear and temporal dynamics can serve as a computing element. Here, we present the first application of physical reservoir computing with plants. In addition to investigating classical benchmark tasks, we show that *Fragaria × ananassa* (strawberry) plants can solve environmental and eco-physiological tasks using only eight leaf thickness sensors. Although the results indicate that plants are not suitable for general-purpose computation but are well-suited for eco-physiological tasks such as photosynthetic rate and transpiration rate. Having the means to investigate the information processing by plants improves quantification and understanding of integrative plant responses to dynamic changes in their environment. This first demonstration of physical reservoir computing with plants is key for transitioning towards a holistic view of phenotyping and early stress detection in precision agriculture applications since physical reservoir computing enables us to analyse plant responses in a general way: environmental changes are processed by plants to optimise their phenotype.

## Introduction

Plants are composed of a series of complex interconnected elements and modules, arranged at different hierarchical levels^1–4^. This complex organisation enables plants to better deal with biotic and abiotic environmental variations in their surroundings^5,6^. Abiotic factors include water and nutrient availability, air temperature, relative humidity, solar radiation, wind speed, and CO_2_ concentration^7^. These abiotic variables directly drive physiological mechanisms such as photosynthesis, water uptake, transpiration and assimilate translocation^7,8^. Major biotic influences are the occurrence of pests and diseases^9^, but also the (symbiotic) associations with microbes and plant-to-plant interactions^9^. Plant physiology is hence determined by its genetic mark-up, abiotic and biotic factors, along with their interactions. Because of these complex interactions, a plant’s functioning cannot be understood by solely studying the constituting elements separately^10^. Instead, an integrated approach is needed that can take into account the complex interactions between a plant and its environment^11^.

Physical reservoir computing (PRC) is an unconventional computing paradigm that utilises physical substrates for computation, originating from computer science^12,13^. This paradigm entails using a high-dimensional, nonlinear dynamical (physical) system as a computational resource to solve a task. Examples encompass the control of mechanical systems by using a compliant robot body^14^, the processing of optical^15^ and electrical^16^ signals, or quantum reservoir computers^17^. In one particular case, a water bucket was used as a physical reservoir to build a liquid computer that could solve the XOR-problem^18^. The XOR-problem is an elementary binary nonlinear function that equals zero when its one-bit inputs are identical and one otherwise. Furthermore, neural activity in a cat’s primary visual cortex can be used as a reservoir to classify several different visual stimuli^19^. Recent reviews^12,13^ highlight that a wide range of physical media can be used to perform physical reservoir computing, including biological substrates such as (*in vivo*) brain cells^20^ and *Escherichia coli* bacteria cultures^21^. Suitable media for PRC must satisfy multiple requirements, such as high-dimensionality, nonlinearity, fading memory with respect to past inputs, and sufficient separation of targets with respect to recent inputs^12^.

**Figure 1:**
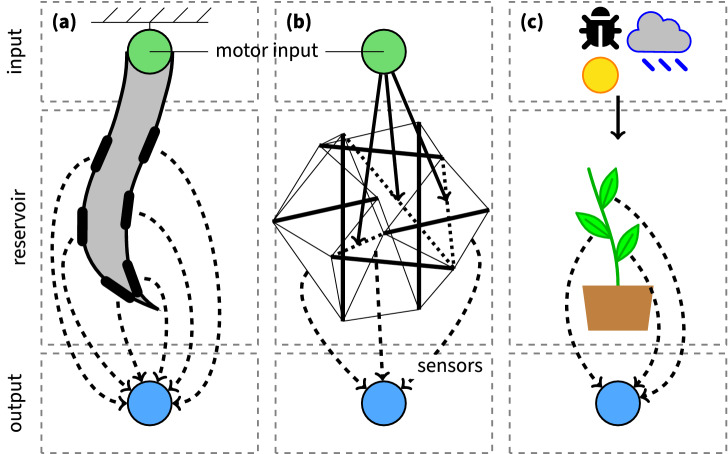
Graphical illustration of physical reservoir computing implementations. (**a**) A soft robotic arm implementation with embedded deformation sensors^29^, (**b**) a robot body built out of a tensegrity structure^30^ (a spring-mass system) that consists of passive and active springs (thin and dotted lines, resp.) and beams (thick lines). These beams have a fixed length and cannot deform, while the springs can contract and/or extend. Force sensors measure the state of the reservoir. (**c**) A plant reservoir computing implementation with biotic and abiotic factors as inputs and plant’s sensors to monitor the plant state.

Similar to the aforementioned physical reservoir computing implementations, plants are high-dimensional nonlinear dynamical systems themselves. Despite the absence of a brain-like organ and their inability to move, plants are capable of reacting effectively to their dynamic environment, just like animals and humans^22^. For example, roots grow by assessing the future acquisition of minerals^23^ and plants possess a memory of previous light incidents, which is used for the optimisation of future light acclimation and optimisation responses^24^. In other words, plants can store and use information of light sum, intensity and day length for several days or more to anticipate changes that might appear in the near future in the environment^25^. These examples illustrate that plants have typical learning behaviour (habituation, priming) and complexly integrated store/recall systems of memory^26^. In fact, a plant continually gathers and updates diverse information about its environment, integrates this with information on its present internal state, and then makes decisions that reconcile its well-being with its environment^27^. Hence, we can consider the plant as a computing unit, able to process multiple signals to provide an integrated response that maximises fitness to the prevailing environmental conditions, as discussed in^28^. A readout of the plant responses combined with a measurement of the plant’s environment can then be used to model its reactions to complex environmental cues such as drought and temperature stress, incident light, disease and pests. This view can be linked to physical reservoir computing with the plant as the physical reservoir, illustrated in Fig. 1. Figure 1a depicts a soft robotic arm made of silicone^29^. The motor is the input, which drives the reservoir. Deformation sensors embedded into the silicone arm or reservoir observe its state. These state observations are combined to solve a certain task (output layer). The tensegrity robot in Fig. 1b behaves similarly. A motor input signal drives the active springs (dotted lines), which cause deformations of the passive springs (thin lines). The beams (thick lines) have a fixed length and define the nonlinear properties of the system. In plant reservoir computing, Fig. 1c, the environmental cues are the input of the (plant) reservoir. Plant sensors are used to characterise the plant’s state. These state observations are combined to solve tasks such as prediction of the eco-physiological parameters or detection of stress. Considering that plants are not designed for computation, it is unlikely that plants can serve as efficient general-purpose digital or analogue computational devices. Still, plant reservoir computing may have substantial impact on the plant science community. It can provide a holistic approach to plant modelling, improve plant sensing and bring new insights into plant physiology. This computational framework offers a general basis that can be used to study plant behaviour, where a plant’s state is the result of its information processing of all incoming environmental and internal signals. Such a view can be applied to plant functioning and development in general instead of focusing on certain plant processes as the result of a specific treatment.

In this study, we demonstrate plant reservoir computing. While former studies have theorised on computing with plants^31^, to the best of our knowledge, this is the first experimental evidence of physical reservoir computing with plants. We show this by observing the plant’s dynamical state with contact-based leaf thickness sensors. We map temporal input patterns from leaf thickness sensors with a simple linear readout function to estimate (i) the environmental conditions, (ii) eco-physiological tasks, and (iii) computational benchmark tasks.

## Results

### Evaluation of the PRC framework for plants

**Figure 2:**
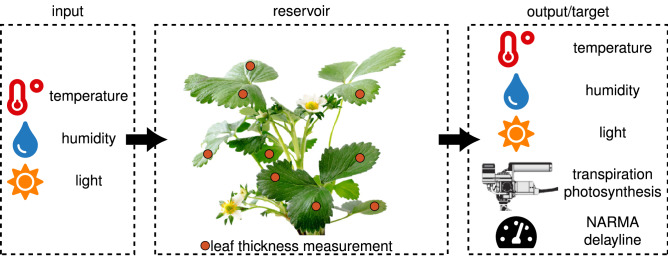
Conceptual overview of the experiments. The three modulated input variables form the input of the plant-reservoir. The reservoir reacts according to current and previous inputs and thus the leaf thickness changes. The leaf thickness is observerd and is considered the output of the reservoir. These outputs are then combined to predict one of the output targets.

To demonstrate the computing properties of plants, we set up a series of experiments on *Fragaria* $\times$ *ananassa* (strawberry) where we monitor key environmental variables and gas exchange activity of the plants, as depicted in Fig. 2. While plants violate the fading memory property over their entire lifetime, we only consider a short period of their mature growing stage when performing the experiments. Each experiment lasts for eight days in a growth chamber. Inside the growth chamber, light intensity, air temperature and relative humidity are modulated, and the plant’s responses are captured using eight randomly placed leaf thickness sensors. All three modulations follow a typical day-night pattern, based on actual weather data where additional randomness was inserted into the light intensity and direction by alternating which set of lamps was turned on without overly affecting the total light intensity. Although these are three main abiotic drivers that influence a plant’s eco-physiology^7^, we consider the light intensity as the main input. The other two abiotic drivers mainly serve to preserve a realistic day-night pattern where plants experience higher temperatures and lower humidity during the day and the inverse at night.

Plants continuously sense their environment and optimise their physiological responses accordingly^9,32^. Consequently, these three modulations are excellent factors that serve as input to the plant reservoir. Leaf thickness is an interesting trait to monitor since it varies rapidly. Leaf thickness is often employed as a proxy for the dynamic plant water balance, which is influenced by variations in environmental variables such as light exposure, temperature, relative humidity and plant-specific factors such as age^33–35^. Consequently, it is a highly informative measure on the status of the plant. Moreover, due to the dependence on local factors, such as light exposure, we expect some heterogeneous behaviour within the plant, which is vital for physical reservoir computing. An additional asset of using leaf thickness for reservoir computing, is the fact that leaf thickness variations persist in mature leaves, without gradually increasing further. However, the measurement clips are also sensitive to temperature fluctuations. Therefore, to validate that the plant is the main source of computation, we also set up a control experiment where the thickness clips are not mounted on a plant. Yet, a plant is placed into the growth chamber to capture real gas exchange data. This negative control is necessary because there might be a complex interaction of the environment and the sensor system that can also have properties resembling a reservoir.

In total, three experiments were conducted in the growth chamber. Each experiment used the same input modulation traces (light intensity, air temperature and air humidity), but the observed traces might differ slightly due to random changes and settling behaviour of the growth chamber. Moreover, three different plants were used to collect physiological data. As a result, the target signals for each of the tasks considered are experiment-specific, although some are very similar. The main objective of these experiments is to provide a proof-of-concept that demonstrates physical reservoir computing with plants. To that end, a limited number of individuals suffices. However, in a next stage we aim to characterise the overall computational aspects of different plant species.

For more information about the experimental setup, we refer to *Materials and Methods*.

### Task definitions

We consider regression problems solely in this study since all the eco-physiological measurements performed are continuous variables. Moreover, it is difficult to reset a plant to the same initial conditions when a new training sample is provided at the input. Three types of prediction regression targets are considered: (i) environmental targets, which also form the input of the reservoir; (ii) photosynthetic rate $P_n$ and transpiration rate *E* as eco-physiological tasks based on the gas exchange data; and (iii) computational benchmarks. An overview is provided in Table 1.

**Table 1:** Overview of considered types of targets: (i) environmental, (ii) eco-physiological and (iii) computing benchmark targets.

Type	Symbol	Description
i	$T_\text {air}$	Air temperature (GC)
i	*h*	Relative humidity (GC)
i	$I_\text {PAR}$	Photosynthetically active radiation (GC)
ii	$P_n$	Photosynthetic rate
ii	*E*	Transpiration rate
iii	$B_\text {DL}$	Delay line of $I_\text {PAR}$
iii	$B_\text {PL}$	Polynomial transformation of $I_\text {PAR}$
iii	$B_\text {NARMA-n}$	NARMA-n based on $I_\text {PAR}$

Growth chamber (GC) signifies variables measured in the growth chamber.

Reconstructing the environmental input of the reservoir is an interesting task to evaluate how the information at the input is retained by the reservoir. Estimating gas exchange activity ($P_n$ and *E*) from leaf thickness is an interesting biologically relevant task that demonstrates practical applications of PRC with plants. Computational benchmarks are computed to evaluate the nonlinear and memory properties of plants on a more theoretical basis. This is done using two tasks: nonlinear auto-regressive moving average (NARMA) and a delay line. The goal of these tasks is to reconstruct a (modified) version of one of the input signals. In both cases, we consider light intensity $I_\text {PAR}$ as the input signal. The NARMA task is a benchmark task often used to evaluate PRC media^29^. This task has a parameter *n* that influences the amount of nonlinearity and memory, higher values of *n* result in more difficult tasks. We use a slightly modified version such that the memory dependencies operate at the minute scale. Consequently, we increased the memory dependency of the task. This was done because otherwise, the time-dependencies were too extensive, resulting in too much smoothing and even stability issues for large values of *n*:

1 $$\begin{aligned} y(t+1) =\alpha y(t) + \beta y(t)\left( \sum _{i=0}^{n-1}y(t-60i)\right) + \gamma x(t-60n+1)x(t) + \delta \text {.} \end{aligned}$$

The parameters $\alpha$, $\beta$, $\gamma$ and $\delta$ are chosen as 0.3, 0.05, 1.5 and 0.1, respectively^29^. We do not consider general-purpose tasks such as MNIST digit recognition, language classification or a 2-bit XOR task, as is demonstrated in other PRC research^15,36^. In the context of reservoir computing with plants, we do not consider these tasks as relevant since plants are unlikely to outperform conventional computing devices for such tasks. Instead, we focus on plant-specific tasks that are more relevant with respect to future applications in plant eco-physiology and phenotyping.

Since models are not transferable between experiments, we estimate the variability due to sensor placement by selecting seven out of eight sensors. Since individual plants might also have considerably different dynamics, we repeated the experiments for two strawberry plants.

### Target evaluation metric

We use normalised mean squared error (NMSE) as an error metric since it is used in many reservoir computing papers and enables inter-task comparisons. An NMSE score of 1.0 corresponds to a mean-prediction, which can be considered a baseline and 0.0 corresponds to a perfect prediction:

2 $$\begin{aligned} \text {NMSE} = \frac{1}{N} \sum _{t=0}^{N-1} \frac{(y(t)-{\hat{y}}(t))^2 }{\text {var}(y)} \text {.} \end{aligned}$$

### Evaluation of the reservoir performance for biologically relevant tasks

**Figure 3:**
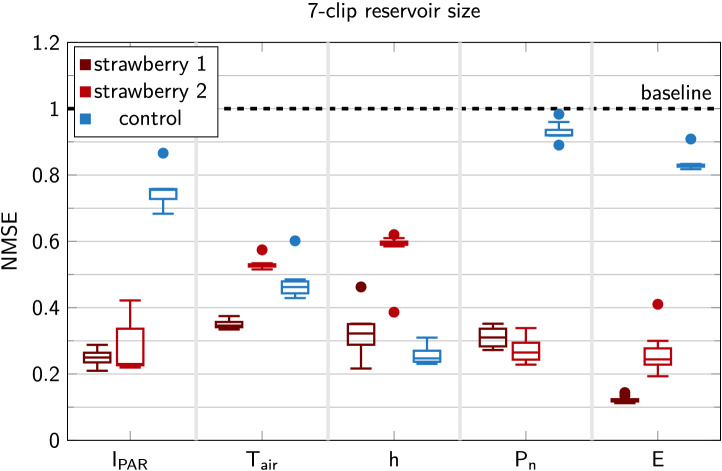
Overview of prediction performance for two different strawberry plants and control using boxplots. The boxplots visualise the effect of different samplings: in each of the samplings, seven out of eight clips are used as reservoir readouts. This allows us to estimate the variability of the random sensor placement. The thickness clips in the control experiment are not mounted on a plant or other material. Whiskers are drawn within the 1.5 interquartile range value from the first and third quartiles. Dots represent outliers that fall outside this range.

Initially, we focus on the biologically relevant tasks. These are the tasks from categories (i) and (ii) (Table 1). Figure 3 visualises the performance using boxplots. Plants outperform the control experiment for $I_\text {PAR}$, $P_n$ and *E*, while the control is better at computing $T_\text {air}$ and *h*. This result is not unexpected since thickness clips are sensitive to temperature fluctuations. A calibration was performed, but due to nonlinear effects, the model is still able to reconstruct $T_\text {air}$ and *h* better in the control experiment.

We also observe that considerable variation between plants may exist: for instance, strawberry 2 is slightly better at estimating $P_n$ than strawberry 1, while the inverse is true for *E*, yet performance for $I_\text {PAR}$ is similar. These differences are probably due to the measurement technique applied for capturing $P_n$ and *E*, which are monitored for one specific leaf. Consequently, there can be a considerable difference between the selected leaf and other leaves, while $I_\text {PAR}$ is an integrated measurement, performed on the same location in both experiments and independent of the plant.

**Figure 4:**
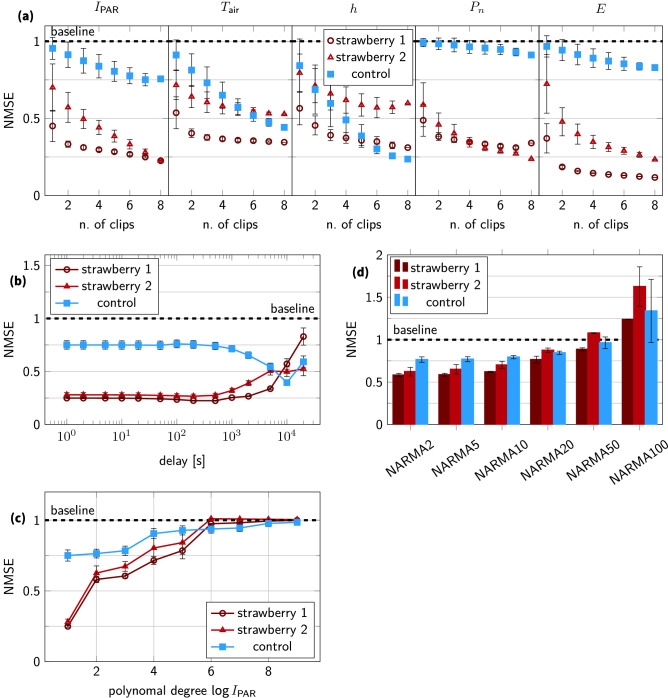
Analysis overview of the reservoir properties and benchmark tasks. (**a**) Depicts the effect of the number of clips (or readouts) on the task performance for environmental and biological tasks. (**b**) Depicts the effect of a delay in the $I_\text {PAR}$ signal prediction task. (**c**) Depicts the polynomial transformation of $I_\text {PAR}$ benchmark task. (**d**) Shows different NARMA tasks, with increasing difficulty. The NARMA benchmark task uses light intensity data $I_\text {PAR}$ as input for $n=\{2,5,10,20,50,100\}$. Error bars in each of the subfigures indicate the standard deviation.

This analysis with two individuals is insufficient to characterise the variability for each of these tasks in strawberry. Yet, the boxplots in Fig. 3 depict the variability we can expect for each individual plant. This is also of importance to assess the number of sensors needed, which is also linked to Fig. 4a.

Analysis of the correlation of each experiment’s inputs (leaf thickness values) and outputs (environmental conditions and eco-physiological variables) indicates that the correlation between input and output is much lower in case of the control experiment than the plant experiments. On average, the correlation between input and output are 0.31 (± 0.21), 0.76 (± 0.20) and 0.67 (± 0.22) for control, strawberry 1 and strawberry 2, respectively. We always report absolute values of the Pearson correlation coefficient. Whenever we report a mean value of a set of correlation coefficients, than the standard deviation is included between brackets. There is considerable correlation between the environmental factors too, especially between $T_\text {air}$ and *h* for all experiments (0.84 and 0.83 and 0.84 for control, strawberry 1 and strawberry 2, respectively). While this is undesirable, this is the result of applying a realistic day-night pattern. Indeed, during the day, light intensity and temperature slowly increase in the morning and decrease as nightfall approaches, while the inverse typically happens for relative humidity. Applying a realistic day-night pattern is important since many internal plant processes are controlled based on an internal circadian clock^9^.

### Evaluation of the reservoir properties

It is vital to study the characteristics of the reservoir to stimulate the development of better plant-based reservoirs and to improve data extraction efficiency. To this end, we evaluate the performance on three benchmark tasks: a delay line, polynomial fit and the NARMA task. Moreover, the reservoir size is also a key parameter to investigate.

The effect of the number of readouts of the reservoir (i.e. the amount of thickness clips) on the environmental and benchmark tasks is depicted in Fig. 4a. As expected, performance increases if we increase the number of observations. Furthermore, the variability decreases because a larger set of sensors is able to capture the dynamics present in the reservoir. However, the performance gain due to increasing readout size also decreases as it increases. This is expected since the larger readout size provides a complete representation of the reservoir dynamics.

We investigate nonlinearly and memory performance in Fig. 4b–d. In Fig. 4b, we initially observe that the NMSE value remains constant for all three experiments as the delay increases on the $I_\text {PAR}$ signal. This is due to the high correlation in leaf thickness among nearby time points. We also note that performance is slightly improved at delays of 500 s and 200 s for strawberry 1 and 2, respectively. As the delay on $I_\text {PAR}$ increases further, performance decreases. Plants perform better than the control, but there is also variation between plants. Peculiar is a drop of the control to 0.4 at 10,000 s. This is an artefact and the result of the temperature dependence of the clips. NARMA is a complex nonlinear task that can have long-lasting dependencies on the past (Eq. 1). As a result, it is an excellently combined task to evaluate the reservoirs. The performance for nonlinear transformations of $I_\text {PAR}$ is depicted in Fig. 4c. The performance quickly degrades as the amount of nonlinearity increases. Strawberry 2 is slightly less performant than strawberry 1. Both reach the baseline for a polynomial degree of 6, when results are similar to those from the control experiment. NARMA tasks with $n=2$ to $n=50$ are depicted in Fig. 4d. The NARMA task is based on the light intensity $I_\text {PAR}$. Plants are better at solving this task than the control experiment. However, both are not very performant on the task since NMSE values are always near or above 0.5. This is also not surprising since plants are not well suited for general-purpose computation. Yet, it is interesting that small values of *n* perform similarly, which is due to the relatively slow variation of leaf thickness (see also Fig. 4b).

## Discussion

In this study, we demonstrate physical reservoir computing on strawberry plants. We show experimentally that plants outperform a control setup for non-trivial tasks such as light intensity $I_\text {PAR}$, transpiration rate *E* and photosynthesis rate $P_n$. Moreover, we also investigate performance on common benchmark tasks such as NARMA-10 and a delay line. In this discussion, we first match our results with literature. Second, we highlight current limitations and future improvements to plant PRC. Finally, we spotlight applications and the broader impact PRC with plants can have on the plant science community.

Literature reports that a significant negative correlation exists between leaf thickness and transpiration rate *E*^37^, explaining why predicting the latter is the best performing task for both strawberry plants in Fig. 3. Though studies on multiple species investigated the correlation between photosynthetic rate $P_n$ and leaf thickness, none have reported significant results^37,38^.

The unexpected drop of NMSE in the curve for the control experiment in Fig. 4b is the result of the correlation between the light intensity $I_\text {PAR}$ and air temperature $T_\text {air}$. This correlation arises due to three effects. First, there is limited correlation between air temperature $T_\text {air}$ and most thickness clips of the control experiment (except for $x_3$). Correlation values range from 0.0 to 0.64 with an average correlation of 0.31 (± 0.22). Though combined, a set of clips is still good at predicting the air temperature (see Figs. 3, 4a) for the control experiment. Second, the correlation between air temperature $T_\text {air}$ and light intensity $I_\text {PAR}$ is maximal at a delay of 4600 s. Consequently, the train error is lowest for a delay of 5000 s and the test error is lowest for 10,000 s for the control experiment. The mismatch between train and validation error is probably due to the model overfitting the data at a delay of 5000 s. At an increased mismatch at a delay of 10,000 s, the model might generalise better. Therefore, the test error is minimal. Correlation matrices are depicted in the supporting information. Third, there is also the natural correlation arising from using a realistic day-night pattern. The temperature is naturally higher during the day due to strong solar radiation and colder at night due to the lack thereof.

Plants show best performance for eco-physiological tasks. This is not unexpected since these tasks are intertwined with their physiology. Moreover, strong couplings exist between plant-processes such as stomatal conductivity, transpiration rate, photosynthesis and leaf thickness due to common driving elements (e.g. water potential and light availability)^35^. The observation that domain-specific computers are better at solving problems within their domain has already been reported in literature. For instance, is has been shown that a soft silicone arm-based computer^39^ and a vortex computer^40^ can outperform conventional machine learning techniques.

For the benchmark tasks, it is essential to compare with other PRC substrates. However, comparing NMSE values from Fig. 3 with other substrates is not straightforward. On the one hand, there are many substrates specifically designed for reservoir computing, such as silicon photonics and memristor chips^15,36^. These substrates perform better on benchmark tasks. For instance, for the NARMA-10 task, photonic reservoirs have NMSE values of 0.035^41^ and for the Santa-Fe time-series prediction task, NMSE values of 0.06 are reported in literature for photonic reservoirs^42^. However, a plant is optimised for fitness, not as a medium for computing^43^, resulting in low or fast degrading performance in Fig. 4c, d. Moreover, many studies mainly focus on simulations since creating a physical reservoir is often time-consuming and expensive, especially if integrated circuits need to be designed. On the other hand, other studies that work with biological media exclusively focus on classification tasks^21,44–47^, a problem distinct from regression. We opted to study regression tasks since these are more relevant from a plant eco-physiological point of view. Additionally, biological signals are also inherently noisy^48^. This noise is difficult to filter given that the reservoir studied here has only up to eight state observations. Despite these limitations, this study is a pivotal first step towards reservoir computing with plants.

Often, the effect of the reservoir size is studied in literature^15,36^, but this is more difficult for plants. Isolating a part of a plant and maintaining its growth as though it was still part of a larger entity is not possible. An integrated perspective is thus necessary. As a result, we study the number of observation points (or readouts) of the reservoir. The number of readouts also greatly affects performance (i.e. lower NMSE values for larger numbers of observations), as indicated in Fig. 4a. This illustrates that an increased number of observations and PRC can improve the prediction accuracy of transpiration rate *E* and photosynthetic rate $P_n$ beyond what is possible using a single sensor. In literature, this effect has also been reported, as well as the saturation effect for as the number of readouts increases^49^. Increasing the number of readouts has an effect on the fraction of observed dynamics. Full observability is not possible for plant-based reservoirs, even if the leaf thickness variation of each leaf is characterised. While short-term leaf thickness variations are a good proxy for plant water status dynamics, there are many more unknown factors such as hormones, metabolism, nutrient and carbon dynamics. These are also part of the reservoir but not directly quantifiable using leaf thickness measurements, although correlations will exist with leaf thickness because merely all plant processes are impacted by the plant’s water status^35,50^.

The results presented in Figs. 3 and 4 are promising. Better sensor technology and calibration can likely reduce unwanted effects due to the sensor-environment interaction and improve signal extraction. Alternative sensor systems such as biopotential^51^, sap flow^52^ or leaf length^50^ might be better suited for than leaf thickness certain tasks.

We identify three main issues with PRC for plants: the effect of uncontrolled and uncharacterised inputs, non-stationarity of plants and plants do not experience their environment in discrete time. First, plants are sensitive to many signals, including the three environmental variables modulated here, but also chemicals (both airborne and in the soil), mechanical stimulation, electricity, and sound^53^. None of these factors is easily controlled and/or kept constant. As a result, these additional input sources possibly distort the applied input signals^54^. One could argue that the reservoir should be able to cope with these additional variations, but there are also limits to the observable processes using thickness clips. Second, plants are non-stationary entities. They keep on developing^55^ and over time, they violate the fading-memory requirement. This requirement is sometimes also called the echo-state property. By selecting leaf thickness, we avoided drastic short-term non-stationary variations in the plant, since leaf thickness in mature leaves saturated. Consequently, the echo-state property is approximately met for the duration of the experiment given that leaf thickness in this stage oscillates around this saturated value based on environmental conditions. However, on a larger timescale, the system remains non-stationary since leaves eventually die off. As a result, online unsupervised learning algorithms are required to create a readout mechanism that is able to cope with changes in the reservoir. One way this can be tackled is using reward-modulated Hebbian learning^56^. In this regard, physical reservoir computing with plants will never be identical to more classic reservoirs such as memristors. This research can form a starting point towards a further generalisation of physical reservoir computing to non-stationary systems. In literature, an extension of the information processing capacity from time invariant to time-variant systems was recently proposed^57^. This masks an important first step towards a generalised computing framework for time-variant systems. Third, plants continuously sense environmental changes and act accordingly. Hence, they do not respond in discrete time. In this study, we did not investigate the implications this has on the reservoir performance and the observed dynamics.

After all, plants are complex integrated systems that contain many coupled processes that occur at different timescales. For instance, photons are absorbed by chlorophyll molecules within 1 fs, whereas chlorophyll fluorescence is emitted in 1 ns after photon incidence^9^. More integrated processes such as stomatal opening and closure respond in the order of 20 s after a change in illuminance. Hydraulic functioning (e.g. water transport) changes in the range of seconds to minutes, whereas organ growth rates vary in the order of minutes to hours^7,55^. Consequently, a plant-based reservoir also operates at these timescales though not all of them are observable using leaf thickness sensors. Alternative sensor technologies can be applied to study other processes, though experimentally evaluating them is time consuming and slow. As such, one could rely on plant models with sufficient details such as functional structural plant models (FSPMs)^58^ to investigate suitable reservoir-like plant processes and evaluate sensor technologies *in silico*. Employing advanced plant models can not only provide information on suitable processes at different organisational levels (and thus also sensing technologies), but also on the timescales^59^ as which we can perform reservoir computing.

While the experiments presented here are mainly theoretical, they may result in practical applications in future work. Treating a plant as a computing entity can help to generalise plant behaviour and provide essential context to physiological studies. Each trait exhibited by a plant can be viewed as the result of the complex interaction between environmental queues and plant behaviour. Essentially, a plant can be viewed as a computational unit that analyses the incoming environmental signals and optimises its physiology accordingly.

Considering the plant as an information processing unit leads to a more holistic view of plant phenotyping, integrating the effect of plant responses over all environmental cues, thus stepping away from only considering specific aspects. While the work presented here is fundamental research on PRC with plants, applications in breeding and phenotyping can already be identified. For instance, in Fig. 4b, we observed a slight dip around 200–500 s. As a result, there might be a lag between a change in light intensity and the resulting difference in leaf thickness^60^. This dip may imply a time lag of 200–500 s between acclimation of the leaf thickness and the changing light intensity. This lag can signify a suboptimal response of the leaf to the fast-changing light intensity. Quantifying, studying and improving such relationships (i.e. reducing the time lag) is especially relevant for plants in the field since they are subject to fast-changing light intensities. Though optimising this dynamic behaviour of plants is often ignored and could even be more important than static performance^61,62^. Reservoir computing can provide the means to characterise this mismatch. More generally, plant reservoir computing may help to identify plants that are able to respond more appropriately to environmental drivers, thereby extending the phenotyping capabilities for breeding. In a broader phenotyping context, PRC can provide a means to interpret plant responses. It allows for the interpretation of experiments in which many environmental factors fluctuate instead of varying only a few. This is a more realistic setting that can help us better understand how plants react and interact with their surroundings in ecological and agricultural settings^11^. Yet, applications need not be limited to breeding and phenotyping. By means of PRC, plants can become active participants in the control loops of agricultural systems. This is in stark contrast to following a predefined trajectory or relying on (in)direct measurements or manual assessment to detect sub-optimal growing conditions. Consequently, stress experienced by plants can be rapidly discovered and actions for rectification of the stress cause can be taken earlier. PRC with plants can thus form an alternative to conventional machine learning approaches that are being introduced in agriculture. For such agricultural systems, extensive domain knowledge and datasets are typically needed to achieve good results^63^. By investigating a more plant-centric method, we hope an alternative approach will arise that avoids such problems and is better able to bootstrap itself though global rewards for instance in the case of Hebbian learning^56^.

To summarise, in this work, we presented—to the best of our knowledge—the first application of physical reservoir computing with plants, more specifically strawberry (*Fragaria* $\times$ *ananassa*). We investigated several types of tasks, including environmental, eco-physiological and benchmark tasks. The results indicate that plants are not suited for general-purpose (digital) computation but are potentially highly interesting for plant-specific tasks and applications in phenotyping. Plants are best at solving eco-physiological and environmental tasks, more specifically transpiration rate *E*, photosynthesis rate $P_n$ and light intensity $I_\text {PAR}$.

## Materials and methods

### Experimental setup

**Figure 5:**
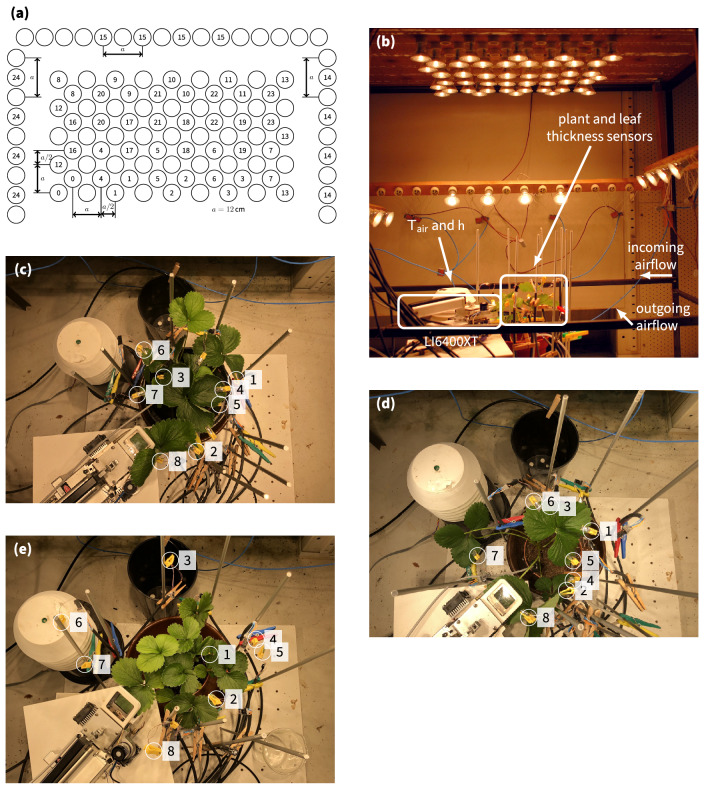
Overview of the three experiments and leaf thickness sensor locations. The clip number of each sensor is depicted in the figure. (**a**) Lamp grid on the top and sides of the frame. Circles indicate a single LED lamp and the numbers indicate the group to which this lamp belongs. Empty sockets are circles without a number. The left, right and top rows (groups 24, 14 and 15 respectively) were mounted on the sides and help to create directional lighting. (**b**) Entire setup inside the growth chamber. Different measurement instruments are indicated as well as the airflow inside the growth chamber. (**c**)–(**e**) Images depicting the setup at the end of each experiment: (**c**) strawberry 1, (**d**) strawberry 2 and (**e**) the control experiment. Carefully observe that the clips are not mounted on the plant in (**e**). There are also two unlabelled clips visible in the images, these were discarded due to sensor failure.

To evaluate the computational properties of plants, we set up a series of experiments in a growth chamber at ILVO (Melle, Belgium). The growth chamber modulated the light intensity, temperature and relative humidity based on a predefined trajectory. These modulations were based on weather observations in Melle, Belgium. The growth chamber had a size $1.45\,\text {m} \times 0.77\,\text {m} \times 1.45\,\text {m}$ (height $\times$ depth $\times$ width) (BIOCLIM 1600 US, Weiss Technik, Reiskirchen, Germany). A custom-built frame of 1.00 m $\times$ 0.70 m $\times$ 1.10 m (height $\times$ depth $\times$ width) was inserted into the chamber. Lamps were mounted on the top and three sides of the frame for illumination. We used 57 LED lamps (PARATHOM DIM PAR16 50 36D OSRAM GmbH, Munich, Germany). The LED lights were arranged in groups that could be individually turned on and off. A detailed overview of the grid is depicted in Fig. 5a, while the entire setup is depicted in Fig. 5b.

The modulation of the environmental conditions (light intensity, temperature and relative humidity) was performed using the Gloxinia sensor platform^64^. This platform also performed sensor readout. Each experiment featured a digital light sensor (APDS9306, Broadcom Inc., San Jose, California, USA), a relative humidity and temperature sensor (SHT35, Sensirion AG, Switzerland) and leaf thickness clips (AH-303, AgriHouse, Berthoud, CO, USA). Furthermore, a single mature leaf was inserted into a transparent leaf chamber of the LI-6400XT photosynthesis system (LI-COR, Lincoln, NE, USA) to acquire gas exchange measurements (transpiration and photosynthesis). The Gloxinia system also controlled the sampling time steps of the LI-6400XT, using a custom circuit that was connected to the manual sample button on the infrared gas analyser (IRGA). Each leaf thickness sensor was sampled every second, while the gas exchange measurement had a sample period of 3s. Faster sampling was not possible due to a limitation of the device.

To ensure that the conditions in the leaf chamber were as similar as possible to those of the rest of the plant, we used an external temperature probe (Vaisala 50Y, Vaisala, Helsinki, Finland) to recreate the outside temperature inside the leaf chamber. This also prevented the chamber from heating up due to the incoming radiation. Moreover, the gas inlet was also positioned close to the plant for maximum consistency. Figure 5b depicts the setup for a strawberry experiment. An image of each experimental setup is provided for each experiment in the dataset. Individual sensor locations are also indicated in Fig. 5c–e using a digit.

To simulate a variable light environment, we varied the light pattern semi-randomly. The number of active lights was static and determined by the PAR value of the weather data (i.e. a proportional number of lights was activated with respect to the maximum light intensity). The set of lights that is turned on was drawn from a uniform distribution. This was updated every 5 minutes.

### Data preprocessing

**Figure 6:**
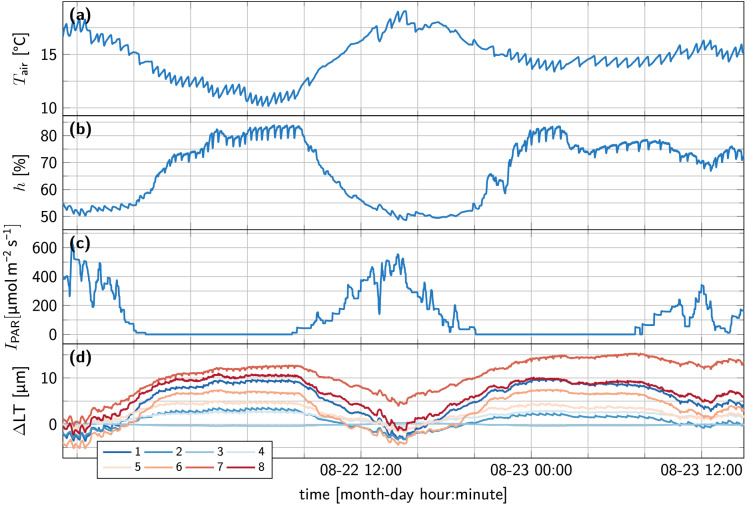
Visualisation of the recorded time series data from the first strawberry experiment. (**a**–**c**) visualise the environmental conditions in the growth chamber and (**d**) visualises the deviation in leaf thickness from the start of the experiment ($\Delta$LT).

Time traces for two days of strawberry 1 are depicted in Fig. 6, including the environmental data and leaf thickness data for all eight clips. The leaf thickness data is depicted as difference from the first sample time point. While the main day-night trend is visible in all eight leaf thickness traces, small differences can also be observed between different clips.

The data from all three experiments was first manually inspected and cleaned to ensure no transient behaviour was included in the analysis. Sometimes the data logging also had to restart due to an error condition occurring because of interference of the high-power and low-power circuits. A restart event resulted in data loss for approximately one minute. Data within this time interval was reconstructed using linear interpolation. The first three hours and last hour of data were also discarded to remove transient effects due to the start or end of the experiment.

Linear interpolation was also used to match the sampling rate of the gas exchange system, leaf thickness and environmental measurements. Unless specified otherwise, data was not processed and/or filtered further.

### Data split and model training

**Figure 7:**
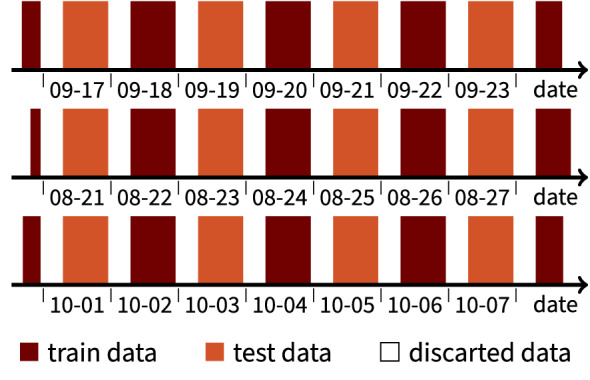
Train/validation and test data split for all three experiments. Top, middle and bottom axes are the control, strawberry 1 and strawberry 2 experiment respectively.

The time-series data generated in the three experiments here are highly correlated. To reduce this correlation, we used a data split into train and test data with interleaving (Fig. 7). Each day was assigned to either the train or test data. Eight hours were discarded between days. This ensures that night-time conditions are not overly represented in the dataset and that there is a decreased correlation between both the train and test datasets. However, because a day-night environmental pattern was followed in the growth chamber, the decreased correlation is limited in time. The cross-correlation is visualised for the strawberry 1 experiment in the supplementary information.

The general idea behind physical reservoir computing is to use the dynamics and memory of the physical substrate for computation. As a result, the learning method needed to fit the target is usually very simple. Essentially, the input is mapped onto a high dimensional space, called a reservoir, consisting of a physical substrate or plant in this study. Because of this high-dimensional mapping, a simple readout mechanism suffices to obtain the desired target from this space.

The simple readout system chosen here, is linear regression with Tikhonov or R2-regularisation^65^. This is a very simple model that converges fast. The Scikit-learn framework was used to train the system^66^. The main equation and loss criterion are:

3 $$\begin{aligned} {\hat{y}}(n) = w_0 + \sum _{i=1}^{8} w_i {\mathbf {x}}(n) \end{aligned}$$

4 $$\begin{aligned} {\mathscr{L}} = \frac{1}{M} \sum _{i=0}^{M-1}(y(i) - {\hat{y}}(i))^2 + \lambda \sum _{i=1}^{8}|w_i|^2 \text {,} \end{aligned}$$

${\hat{y}}(n)$ is the regression prediction output at time point *n* based on the leaf thickness data ${\mathbf {x}} = {[x_1, x_2, \ldots , x_8]}^\text {T}$. The leaf thickness data was re-scaled to have unit variance and a zero mean. The weight coefficients $w_i$ were optimised such that the loss ${\mathscr {L}}$ was minimised for the training data, consisting of *M* samples. $\lambda$ is a hyperparameter that tunes the scale of the weight coefficients and prevents the model from overfitting on the training data.

To optimise the weight coefficients $w_i$ and hyperparameter $\lambda$, a parameter sweep was performed of the hyperparameter $\lambda$ from $10^{-10}$ to $10^{10}$ using logarithmic spacing. For each hyperparameter, the model was optimised, and the best model was selected using a validating dataset. The train/validation data split used a leave-one-out strategy: we use the data of a single day for validation and all the other days for training. This assignment is also permuted such that all days are used for validation. The final choice for $\lambda$ was again optimised using all the training data (including validation data). The final performance was computed on the test data. Code for training and validation are provided with this manuscript.

### Regression tasks

All regression tasks from Table 1 use the measurement data as the target value for ${\hat{y}}$, including the benchmark tasks. Though, the NARMA tasks use a modified version of the light intensity signal $I_\text {PAR}$. $I_\text {PAR}$ is re-scaled to have a zero mean and an amplitude of 0.2. This is done to match the input signal used in other research^29^ and ensure that the output does not diverge since the general form of Eq. (1) is not stable for arbitrary input.

We selected photosynthetic rate $P_n$ and transpiration rate *E* as eco-physiological parameters since these gas exchange measurements are not directly measurable using leaf thickness sensors. The gas exchange sensor device does measure other parameters such as stomatal conductance and leaf temperature, but these are not included since they are highly dependent on temperature, and so are the (leaf) thickness clips.

### Leaf thickness sensor calibration

The leaf thickness sensors used here are sensitive to temperature fluctuations, though they are not equipped with a temperature sensor. As a result, each clip was retrofitted with a thermistor (NXFT15WF104FA2B100, Murata Manufacturing Co., Ltd., Kioto, Japan) that was used for calibration. A linear calibration was performed based on a calibration experiment. During this experiment, the temperature was increased from 10$^{\circ }$C to 30$^{\circ }$C. While it is not necessary for PRC to calibrate the leaf thickness sensors to absolute thickness values, we performed a calibration to obtain fully calibrated sensor values. The clips were calibrated using the calibration card from AgriHouse Calibration Card (AH-300C).

### Plant material

All three experiments used a *Fragaria ananassa* (strawberry) plant of the Elsanta variety. The plants were grown in close proximity in a greenhouse at ILVO (Caritasstraat 39, 9090 Melle, Belgium), thus ensuring that they experienced a very similar growing history. The plants received regular watering to avoid soil water deficit, based on their needs and were grown inside the greenhouse for over one year. All plants are cuttings from the same base plant and were kept free from pests and diseases. No specific treatment was applied to any of the plants. All procedures were conducted according to the ILVO greenhouse safety guidelines for pest control.

## Supplementary Information

Supplementary Information.

## Data Availability

Datasets generated and/or analysed during the current study are available in the Zenodo repository, https://doi.org/10.5281/zenodo.4264624. The data are available on Github using the following identifier: https://github.com/opieters/Leveraging-Plant-Dynamics-Using-Physical-Reservoir-Computing.
