# Supplementary material for: Leveraging plant physiological dynamics using physical reservoir computing
Source: Sci Rep. 2022 Jul 22;12:12594. doi: 10.1038/s41598-022-16874-0 (PMC9307625; doi:10.1038/s41598-022-16874-0)
Supplement: Supplementary file 1 — Supplementary Information. [file 41598_2022_16874_MOESM1_ESM.pdf]

Leveraging Plant Physiological Dynamics Using Physical Reservoir Computing: Supporting Information

Olivier Pieters*, Tom De Swaef[1], Michiel Stock[1], and Francis wyffels[1]

*Corresponding author: olivier.pieters@ugent.be

June 17, 2022

The main text reported only summarised correlation matrix values, the actual correlation matrices are depicted in Figs. S1, S2, and S3.

To provide more insight into the NMSE scores depicted in Fig. 3, we also visualise a time series of the most interesting tasks in Fig. S4. All readouts are used to generate the plots, so no variability data is available. Figure S4a provides an overview of the train and test data, while Fig. S4b zooms in on the grey shaded region of Fig. S4a. This region was not used for training. In the control experiment, the strawberry plant used to obtain the gas exchange data were less active than the other strawberry plants. NMSE values of the test data are also depicted in the upper left corner of each subfigure. From Fig. S4a, we observe that the strawberry reservoirs are more effective (i.e., lower NMSE values), resolving the highs and lows better. In Fig. S4b we observe that strawberry-based reservoirs are better at capturing the dynamic behaviour of each specific eco-physiological task. For example, in the case of I_{PAR}, we see that detailed variation is not captured by the control experiment but is captured by the strawberry experiments. Similar observations can be made for P_n and E. We point out that NMSE also has its limitations; some of the scores for strawberry 2 are close to the baseline of 1.0, similar to the control experiment. Yet, we see that the variation in the target signal is better captured by the plant.

The narrow peaks observed for E in Fig. S4b are an artefact of the measurement device due to slight variations between the measurement channel and reference channel as a result of variable relative humidity.

The correlation for all leaf thickness clips of strawberry 1 is presented in Fig. S5. Indeed, we first see a decreasing correlation until five to seven hours in the experiment, when the correlation increases again. This is due to the day-night pattern of the input variables.

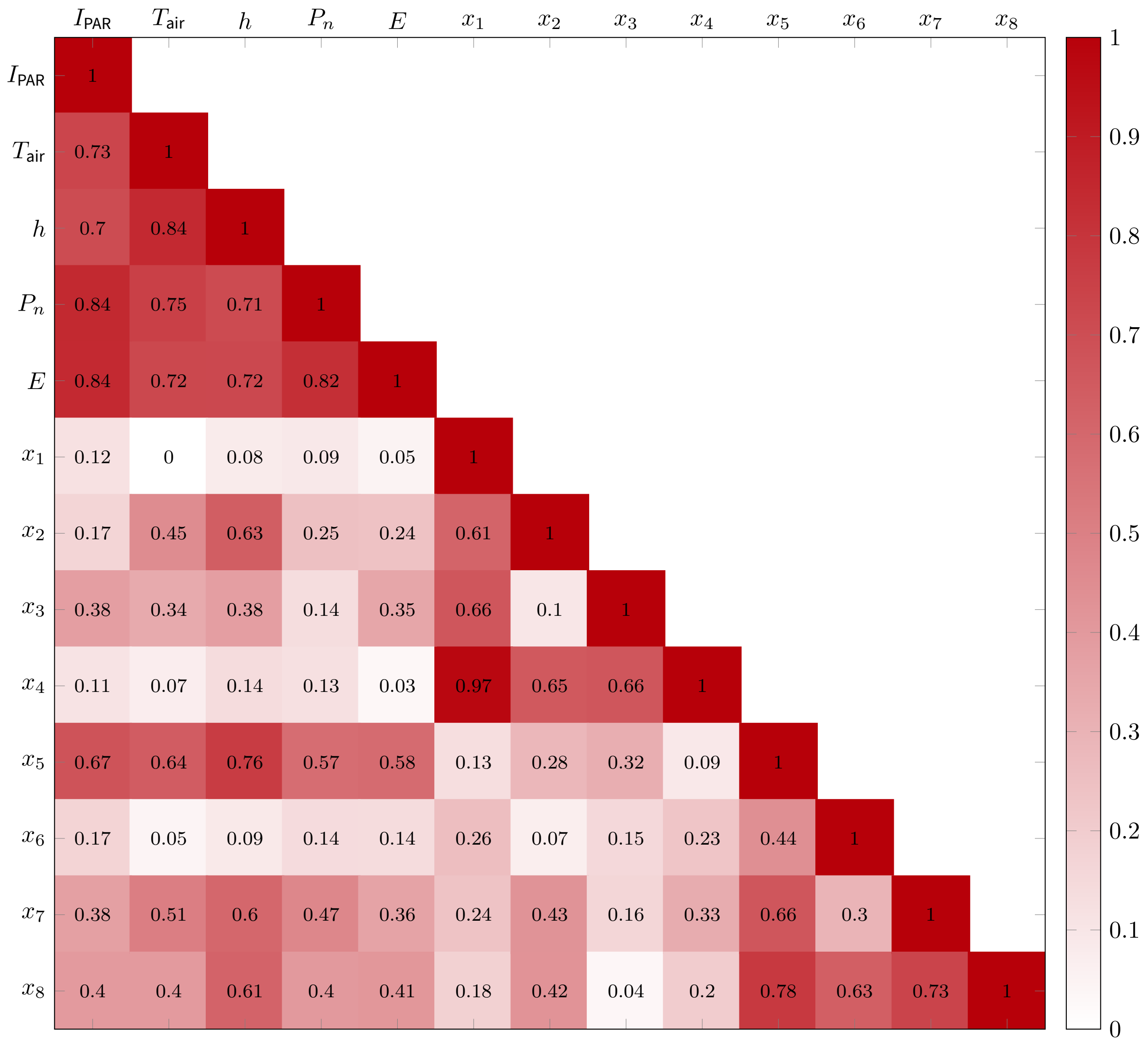

Figure 1: Correlation matrix of the targets (I_{PAR}, T_{air}, h, P_n and E) and (leaf) thickness readouts (x_i) for the control experiment (absolute value of the correlation is depicted).

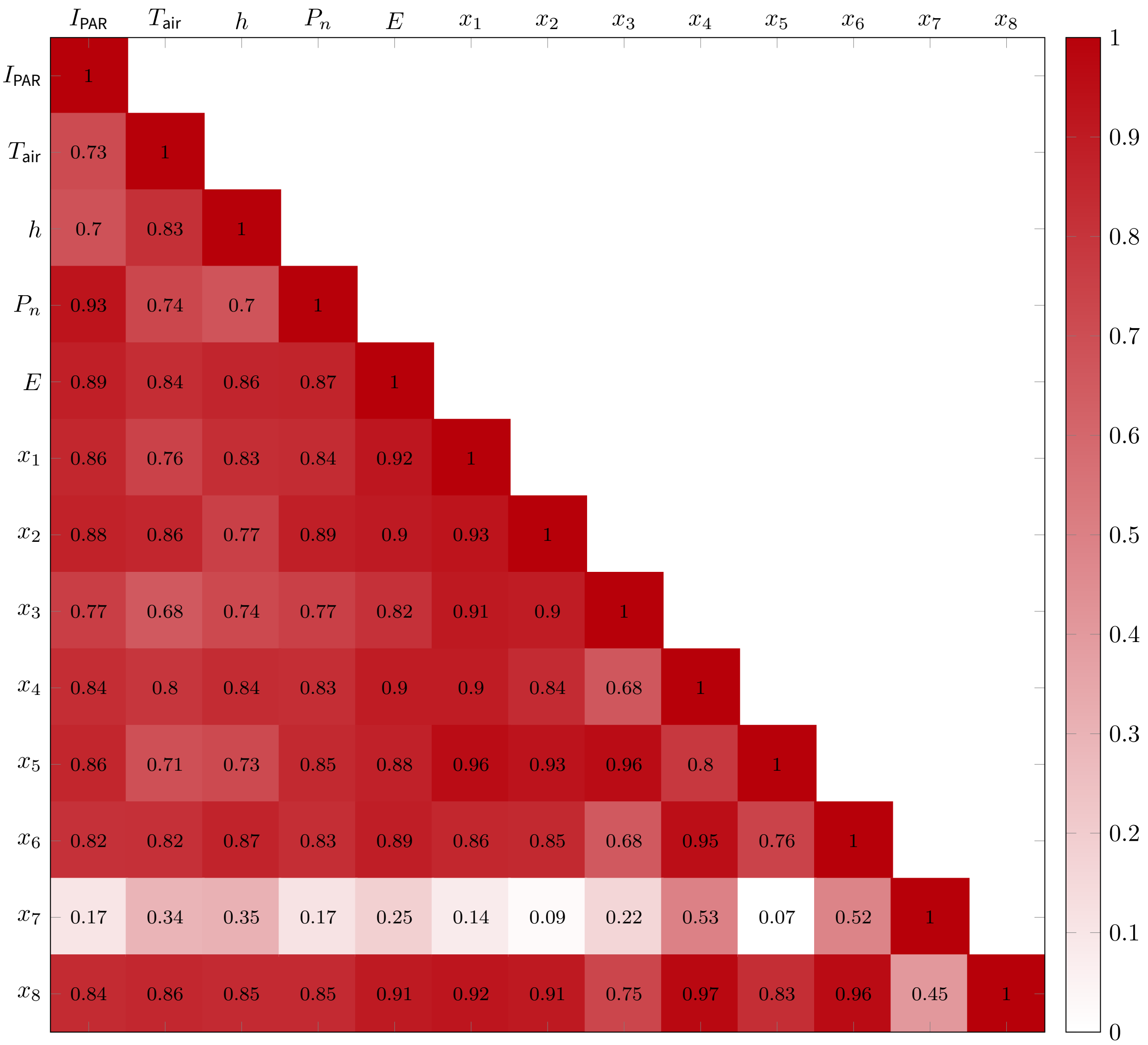

Figure 2: Correlation matrix of the targets (I_{PAR}, T_{air}, h, P_n and E) and (leaf) thickness readouts (x_i) for the first strawberry experiment (absolute value of the correlation is depicted).

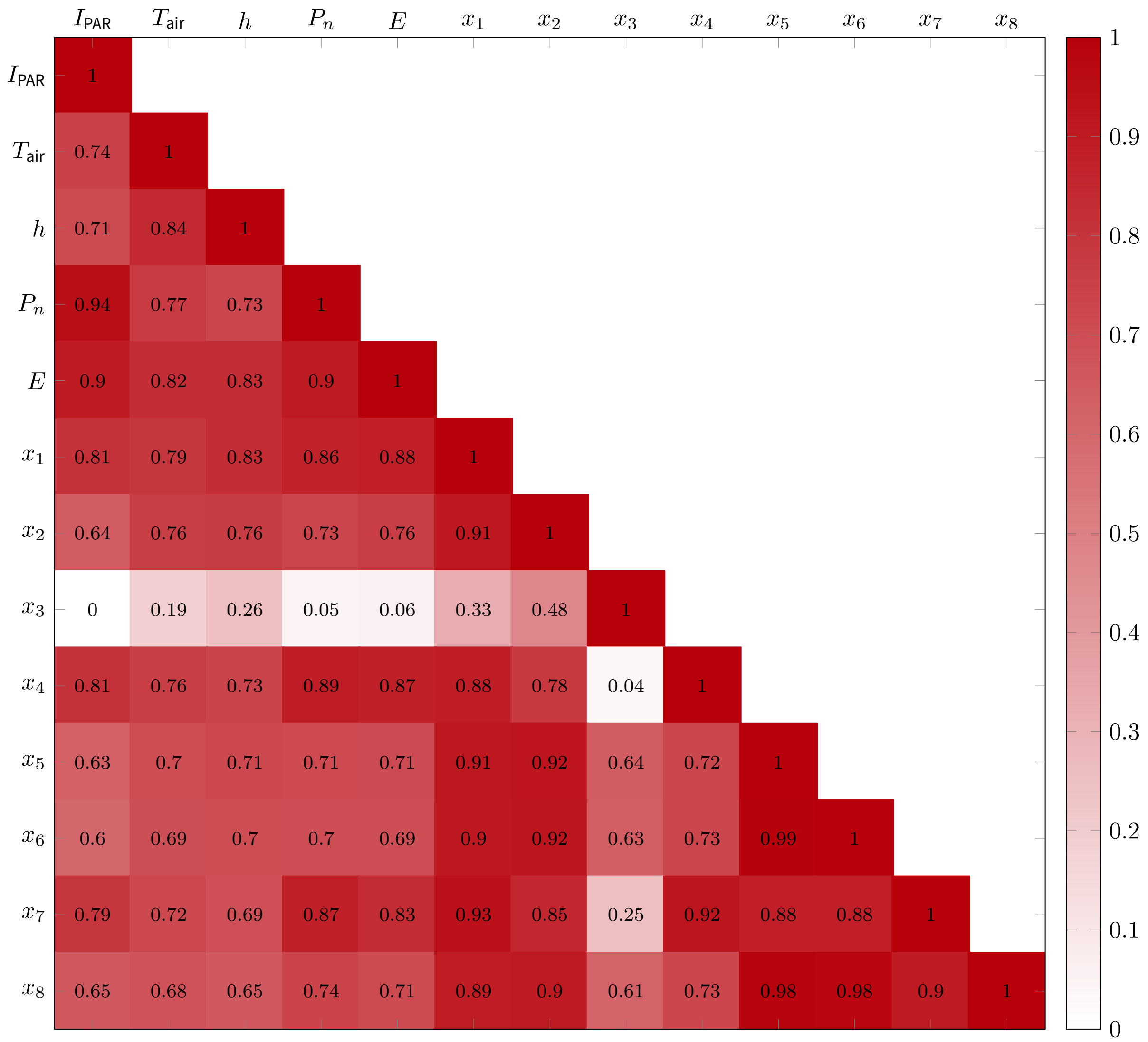

Figure 3: Correlation matrix of the targets (I_{PAR}, T_{air}, h, P_n and E) and (leaf) thickness readouts (x_i) for the second strawberry experiment (absolute value of the correlation is depicted).

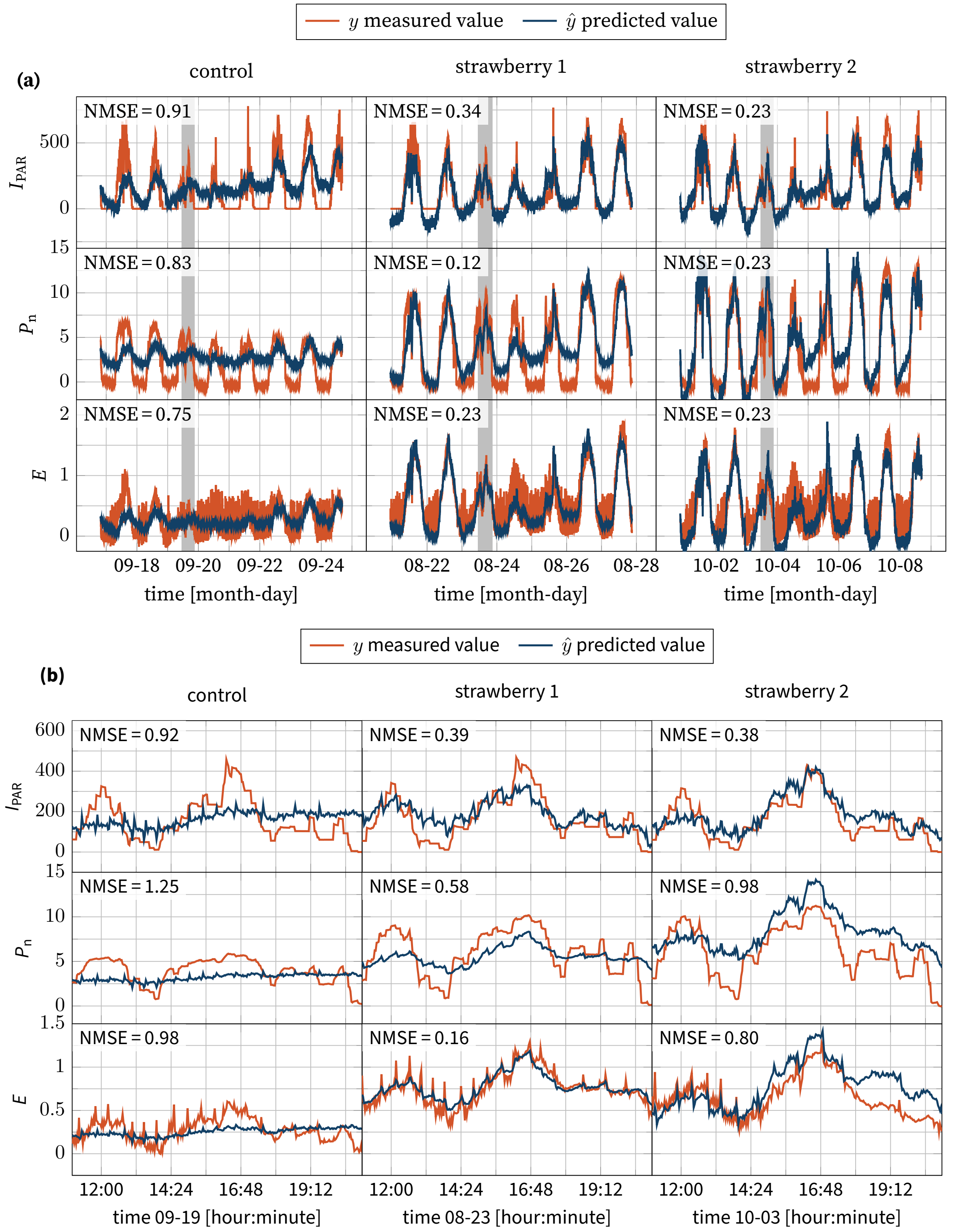

Figure 4: Visualisation of time plot of I_{PAR}, P_n and E. **(a)** visualises the entire dataset. **(b)** zooms in on the grey shaded region of S4a to elucidate more detailed information.

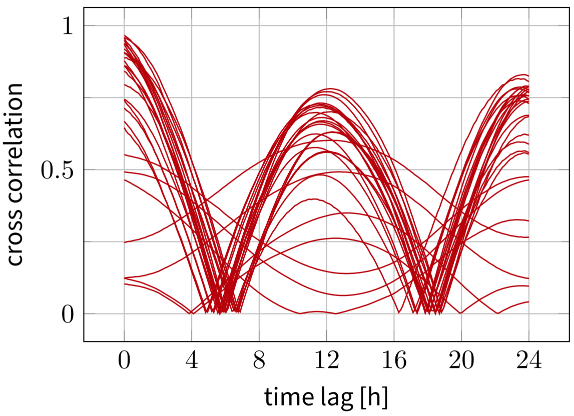

Figure 5: Leaf thickness cross correlation of strawberry experiment 1.
